# Mechanisms of Metabonomic for a Gateway Drug: Nicotine Priming Enhances Behavioral Response to Cocaine with Modification in Energy Metabolism and Neurotransmitter Level

**DOI:** 10.1371/journal.pone.0087040

**Published:** 2014-01-28

**Authors:** Hongyu Li, Qian Bu, Bo Chen, Xue Shao, Zhengtao Hu, Pengchi Deng, Lei Lv, Yi Deng, Ruiming Zhu, Yan Li, Baolai Zhang, Jing Hou, Changman Du, Qian Zhao, Dengqi Fu, Yinglan Zhao, Xiaobo Cen

**Affiliations:** 1 National Chengdu Center for Safety Evaluation of Drugs, State Key Lab of Biotherapy, Sichuan University, Chengdu, Sichuan, China; 2 Analytical and Testing Center, Sichuan University, Chengdu, Sichuan, China; Chiba University Center for Forensic Mental Health, Japan

## Abstract

Nicotine, one of the most commonly used drugs, has become a major concern because tobacco serves as a gateway drug and is linked to illicit drug abuse, such as cocaine and marijuana. However, previous studies mainly focused on certain genes or neurotransmitters which have already been known to participate in drug addiction, lacking endogenous metabolic profiling in a global view. To further explore the mechanism by which nicotine modifies the response to cocaine, we developed two conditioned place preference (CPP) models in mice. In threshold dose model, mice were pretreated with nicotine, followed by cocaine treatment at the dose of 2 mg/kg, a threshold dose of cocaine to induce CPP in mice. In high-dose model, mice were only treated with 20 mg/kg cocaine, which induced a significant CPP. ^1^H nuclear magnetic resonance based on metabonomics was used to investigate metabolic profiles of the nucleus accumbens (NAc) and striatum. We found that nicotine pretreatment dramatically increased CPP induced by 2 mg/kg cocaine, which was similar to 20 mg/kg cocaine-induced CPP. Interestingly, metabolic profiles showed considerable overlap between these two models. These overlapped metabolites mainly included neurotransmitters as well as the molecules participating in energy homeostasis and cellular metabolism. Our results show that the reinforcing effect of nicotine on behavioral response to cocaine may attribute to the modification of some specific metabolites in NAc and striatum, thus creating a favorable metabolic environment for enhancing conditioned rewarding effect of cocaine. Our findings provide an insight into the effect of cigarette smoking on cocaine dependence and the underlying mechanism.

## Introduction

Nicotine and cocaine are two of the most widely abused stimulant drugs in the world. Many surveys have shown that there is a statistical and dramatic association between the use of licit drugs (alcohol or cigarettes) and other illicit drugs [1], [2]. Cigarette smoking acts as a precursor of later illicit drug use [3]. It is reported that 90.4% cocaine users had smoked cigarettes before they began to use cocaine [4]. Behavioral experiments have also proved that nicotine produces an effect on the response to other drugs. For example, nicotine exposure enhances cocaine-induced locomotor activity in mice [5]. Nicotine pre-exposure increases cocaine-induced place preferences in rats [6]. Additionally, several studies have been conducted to explore the mechanism by which nicotine reinforces the response to cocaine. It is reported that nicotine primes the response to cocaine by increasing its ability to induce transcriptional activation of FosB gene through inhibiting histone deacetylase [4]. Nicotine induces dopamine (DA) release in nucleus accumbens (NAc), potentially reinforcing cocaine’s behavioral effects [7], [8]. Nicotine exposure may alter the maturation of central nerve system, and results in a change in reward threshold, thus facilitating the vulnerability for drug dependence [9]. However, these studies only focused on certain genes or neurotransmitters which have already been known to participate in drug addiction, lacking endogenous metabolic profiling in a global view.

Currently, metabonomics has been widely applied in neuropsychiatric research fields, such as motor neuron disease, schizophrenia, Parkinson’s disease and drug addiction [10], [11], [12]. In schizophrenia field, metabonomics findings show systematic changes in pathways of glutamate metabolism and Krebs cycle in the cortex and hippocampus of rats treated with MK-801 [13]. Metabolomics acts as a powerful tool for detecting variations in a range of intracellular compounds upon drug exposure [14]. Unlike genomics, transcriptomics or proteomics, metabonomics shows what indeed happened and could detect the state of multiple metabolites, thus having a potential to identify the related biomarkers. Additionally, Nuclear magnetic resonance (NMR) spectroscopy technique, one of the most commonly used analytical methods in metabonomic study, has been extensively used to investigate the variation of whole metabolites in brain tissues [15]. By using ^1^H NMR-based on metabonomics, some researches showed that neurotransmitter pathways and energy metabolism are affected by addictive substances, such as morphine and cocaine [16], [17].

In this study, we developed a threshold dose model (50 µg/ml nicotine for 7 days, following 2 mg/kg cocaine for 3 days) and a high-dose model of cocaine (water for 7 days, following 20 mg/kg cocaine for 3 days) in mice to explore the mechanism underlying the effects of nicotine on the response to cocaine. We found that nicotine pretreatment dramatically enhanced the behavioral response to subsequent cocaine. Interestingly, with nicotine pretreatment, CPP induced by low dose of cocaine (2 mg/kg) was increased significantly, which was similar to the CPP induced by 20 mg/kg cocaine alone. More excitingly, the modified metabolites in above two models showed considerable overlap, including neurotransmitter, energy substances and membrane components. These results reflect neurotransmitter disturbance, energy metabolism imbalance as well as membrane disruption in NAc and striatum. Our study using behavioral models coupled with global metabolic profiling provides a new insight into the mechanism by which nicotine as a gateway drug reinforces cocaine’s rewarding effects. Moreover, our findings indicate that reprogramming of metabolites may affect cocaine abuse.

## Materials and Methods

### 1. Drugs

Nicotine hydrogen tartrate was purchased from Sigma. Nicotine was dissolved in distilled water and administrated through the drinking water, which was stored in dark bottles and renewed every 2 days. Cocaine hydrochloride was purchased from the National Institute for the Control of Pharmaceutical and Biological Products (Beijing, China), and was dissolved in sterile saline before use.

### 2. Animal Models and Administration

Male C57BL/6J mice (8–12 weeks old) were kept in clear plastic cages with five per cage at under a 12/12 h light-dark cycle in room temperature (21±5°C) with an air change rate of 8–10 changes/hour and a relative humidity of 55±15%, and given food and water ad labium. The animals were acclimatized for 7 days before experiment. This study was carried out in accordance with the guidelines established by the Association for Assessment and Accreditation of Laboratory Animal Care. The protocols were approved by the Institutional Animal Care and Use Committee of the Institute (Protocol number IACUC-S200904-P001). All surgeries were performed under sodium pentobarbital anesthesia, and all efforts were made to minimize suffering. The mice were randomly assigned to 5 groups: drinking water+saline; drinking water +2 mg/kg cocaine; drinking water +20 mg/kg cocaine (high-dose model); 50 µg/ml nicotine+saline; 50 µg/ml nicotine +2 mg/kg cocaine (threshold dose model). Table 1 summarizes the details of the experimental procedure of each group. Cocaine and saline were administrated by intraperitoneal injection (i.p) and nicotine by water drinking. All animal experiments were performed in accordance with the provisions of the Association for Assessment and Accreditation of Laboratory Animal Care (AAALAC).

**Table 1:** Summary of experimental procedure.

Groups	Pretreatment	After-treatment	
	(drink, 7 d)	(i.p., 2 times/d, 3 d)	
		a.m.	p.m.
Saline (control)	water	saline	saline
2 mg/kg cocaine	water	saline	2 mg/kg cocaine
20 mg/kg cocaine	water	saline	20 mg/kg cocaine
Nicotine	nicotine	saline	saline
Nicotine +2 mg/kgcocaine	nicotine	saline	2 mg/kg cocaine
Nicotine +20 mg/kgcocaine	nicotine	saline	20 mg/kg cocaine

### 3. Conditioned Place Preference (CPP)

CPP studies were conducted by using a shuttle box which was composed of two large conditioning chambers and a small central start chamber. One large conditioning chamber had black walls, and the other had white walls. Two groups (nicotine+saline group; nicotine +2 mg/kg cocaine group) were pretreated with nicotine (50 µg/ml) for 7 days continuously; the other three groups (drinking water+saline; drinking water +2 mg/kg cocaine; drinking water +20 mg/kg cocaine) were treated with water for 7 days. On day 8, all mice were placed in the central chamber and allowed to move freely in the apparatus for 30 min to determine the initial preference. Mice with a chamber bias greater than 75% were dropped from studies. On days 9–11 all mice in the study were given one injection of saline and placed in the preferred chamber for 30 min. Four hours later, mice were treated as follows and placed in the non-preferred chamber for 30 min: one injection of saline in drinking water+saline group or nicotine+saline group; one injection of 2 mg/kg cocaine in drinking water +2 mg/kg cocaine group or nicotine +2 mg/kg cocaine group; one injection of 20 mg/kg cocaine in drinking water +20 mg/kg cocaine group. On day 12, CPP test were conducted for all mice. Each mouse was allowed to move freely in all the chambers for 30 min. Time spent in the previously non-preferred and preferred chambers were recorded. Data were analyzed as time spent in the preferred chamber minus time spent in the non-preferred chamber. Each group was presented as mean ± SD, and one-way analysis of variance (ANOVA) followed by Tukey post hoc test was used to determine statistical significance.

### 4. Preparation of Brain Extracts

At the end of CPP test, mice were sacrificed, and brain NAc and striatum (30∼100 mg) were rapidly dissected and stored at −80°C. The preparation of brain samples was based on previous studies [18], [19]. Briefly, the frozen tissue with 0.8 ml of ultrapure water was homogenized by using Branson Sonifier (Japan) for 2 min (200 W) at 4°C, and 0.8 ml ice-cold chloroform was added into the homogenate. The mixture was mixed for 2 min and kept for 10 min on ice, followed by centrifugation at 13,000×g for 10 min at 4°C. The supernatant (∼0.5 ml) was preserved to lyophilize for about 36 hours. After lyophilization, the powder was added into 0.52 ml D_2_O (heavy water) including 0.01 mg/ml sodium (3-trimethylsilyl)-2, 2, 3, and 3-tetradeuteriopropionate (TSP). The supernatant (∼0.5 ml) was shifted into a 5 mm NMR tube for ^1^H NMR detection after centrifugation at 13, 000×g for 5 min at 4°C [20].

### 5. Solution ^1^H NMR Spectroscopy

All the spectral data were obtained on a Bruker-Av II 600 MHz spectrometer (Bruker Co., Germany) at 300 K. A one-dimensional spectrum was acquired by using a standard (1D) Carr–Purcell–Meibom–Gill (CPMG) spin-echo pulse sequence, which suppressed the water signals. The free induction decays were weighted by an exponential function with a 0.3-Hz Gaussian Maximum position 0.1, prior to Fourie transformation.

### 6. Data Reduction and Pattern Recognition Analysis

All NMR spectra were automatically reduced to 440 segments, each with a 0.02 ppm width ranging from 0.2 to 4.6 ppm and 5.1 to 9.4 ppm using MestRe-c2.3 software (http://qobrue.usc.es/jsgroup/MestRe-c). The area for each segmented region was calculated. The region of the spectrum (δ 4.6–5.1 ppm) was removed to exclude the influence of water signal. To account for dilution or bulk mass differences between samples, each spectral intensity data set was normalized to the total sum of the spectral regions following exclusion of the water resonance. The datasets were mean-centered prior to partial least squares (PLS), principal component analysis (PCA), orthogonal signal correction (OSC) analysis by the SIMCA package. Two-dimensional score plots were used to visualize the separation of the samples and the corresponding loading plots were applied to identify the altered contribution to the position of spectra. PCA distinguished the characteristic variable (metabolic signals) or the outlier from the group by statistical method. PLS, a supervised pattern recognition (PR) method, was subsequently applied to enhance this separation. OSC, a spectral filtering method, was applied to optimize the separation. The variable importance (VIP) could inform the important values to separate the cluster and the corresponding loadings for PLS models after application of OSC were applied to identify the altered contribution to the position of spectra that was changed after drug treatment. VIP>1 of multivariate were identified distinguishing metabolites.^ 1^H NMR chemical shifts and assignments of endogenous metabolites were conducted according to the previous literatures and the Human Metabolome Database [21] (http://www.hmdb.ca/), a web-based bioinformatic/cheminformatic resource with detailed information about metabolites and metabolic enzymes. All analyses were carried out by SPSS 11.5, and P<0.05 was considered statistically.

## Results

### 1. Exploring Threshold Dose of Cocaine-induced CPP in Mice

**Figure 1:**
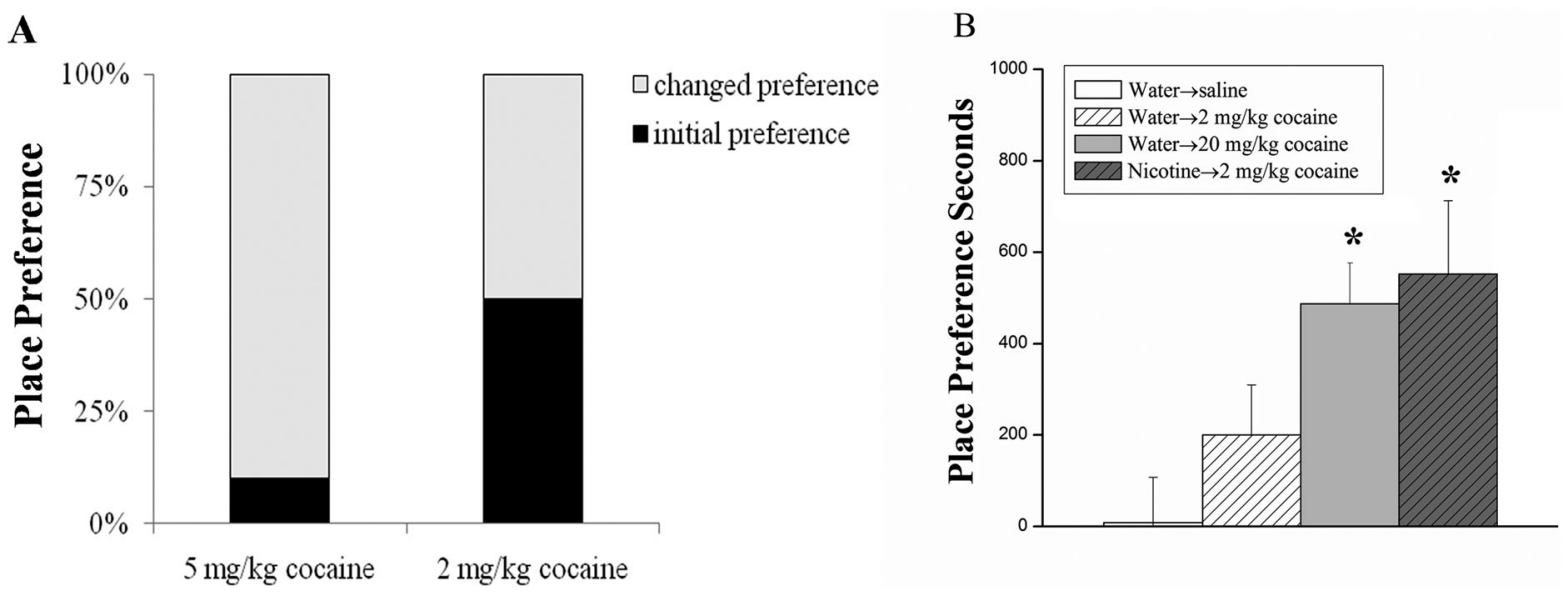
Threshold dose of cocaine-induced CPP is obtained by CPP test and nicotine enhances cocaine-induced CPP (A and B). (A) To explore threshold dose of cocaine-induced CPP, we designed two doses of cocaine (5 mg/kg and 2 mg/kg) to treat mice once a day for 3 days (n = 12 in each group). (B) To detect effects of nicotine on cocaine, four experimental groups were designed (n = 8 for all groups).

Each group spent almost the same time on the initially non-preferred side, indicating that there were no basal differences among groups. To investigate the threshold dose of cocaine-induced CPP in mice, we designed two doses of cocaine (5 mg/kg and 2 mg/kg) to treat mice separately. After administration, it was observed that 90% mice in 5 mg/kg cocaine group (n = 12) spent significantly more time in the non-preferred chamber, and only 10% still spent more time in the initially preferred chamber on the posttest day than the pretest day (P<0.05). However, 50% mice in 2 mg/kg cocaine group (n = 12) moved to the non-preferred chamber for more time, and 50% still stayed in the initially preferred chamber for more time on the post-test day than the pre-test day (P<0.05) (Figure 1A.). These results showed that 2 mg/kg is a threshold dose to the development of CPP induced by cocaine in C57BL/6J mice.

### 2. Nicotine Priming Increases Cocaine-conditioned Place Preference

Cocaine (20 mg/kg dose) has been widely used to induce the development of CPP in mice [22], [23]. Either 2 mg/kg or 20 mg/kg cocaine alone could increase CPP in mice compared with saline control. With nicotine pretreatment mice receiving 2 mg/kg cocaine displayed a 150% further increase of the time in cocaine-paired chamber, which was very close to the time of 20 mg/kg cocaine group (Figure 1B.). These results indicated that nicotine priming can enhance behavioral response to cocaine.

### 3. NMR Spectra and OSC-PLS Analysis

**Figure 2:**
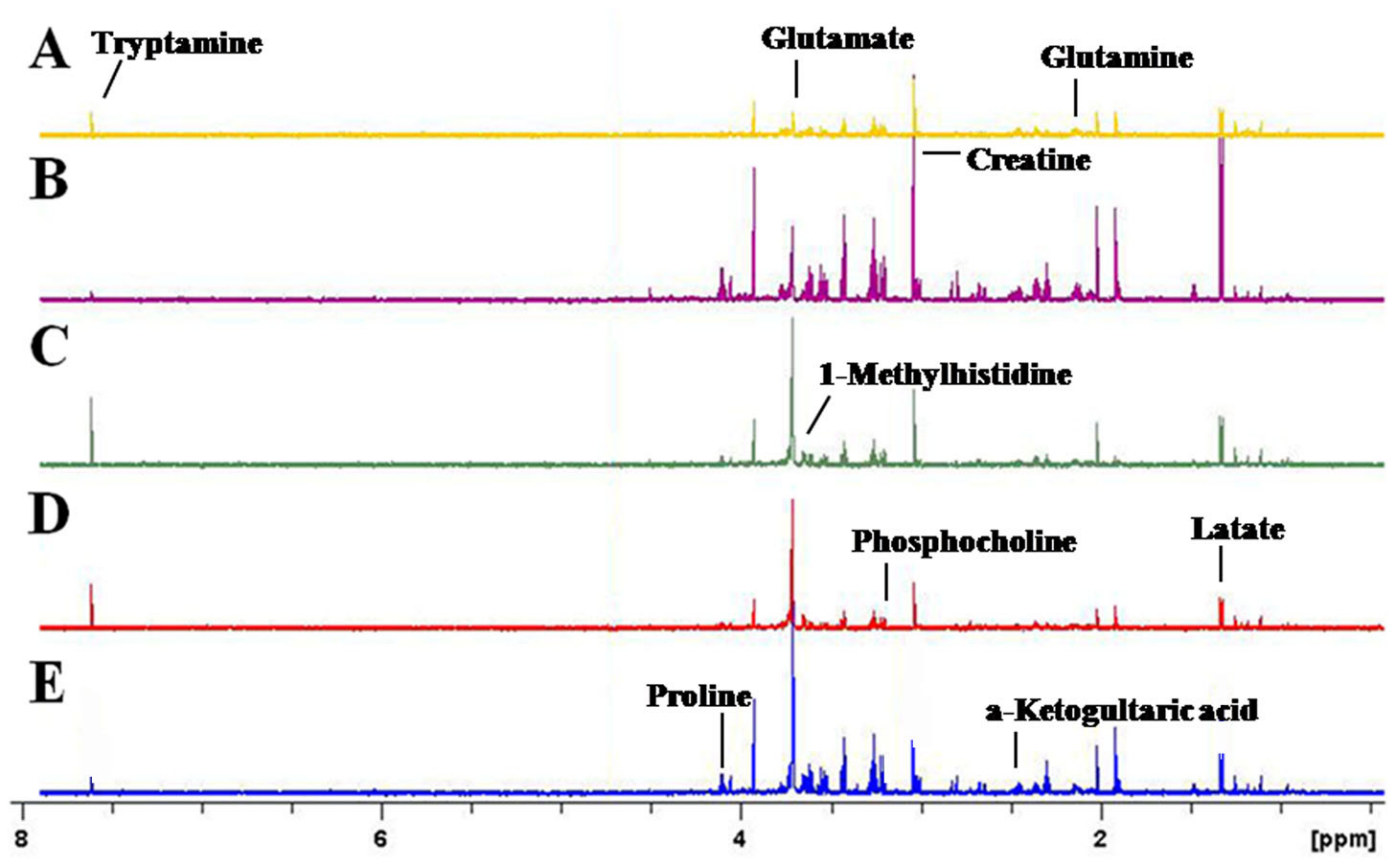
600 MHz CPMG ^1^HNMR spectra of the NAc from mice. (A) control; (B) 2 mg/kg cocaine; (C) 20 mg/kg cocaine; (D) nicotine; (E) nicotine +2 mg/kg cocaine.

**Figure 3:**
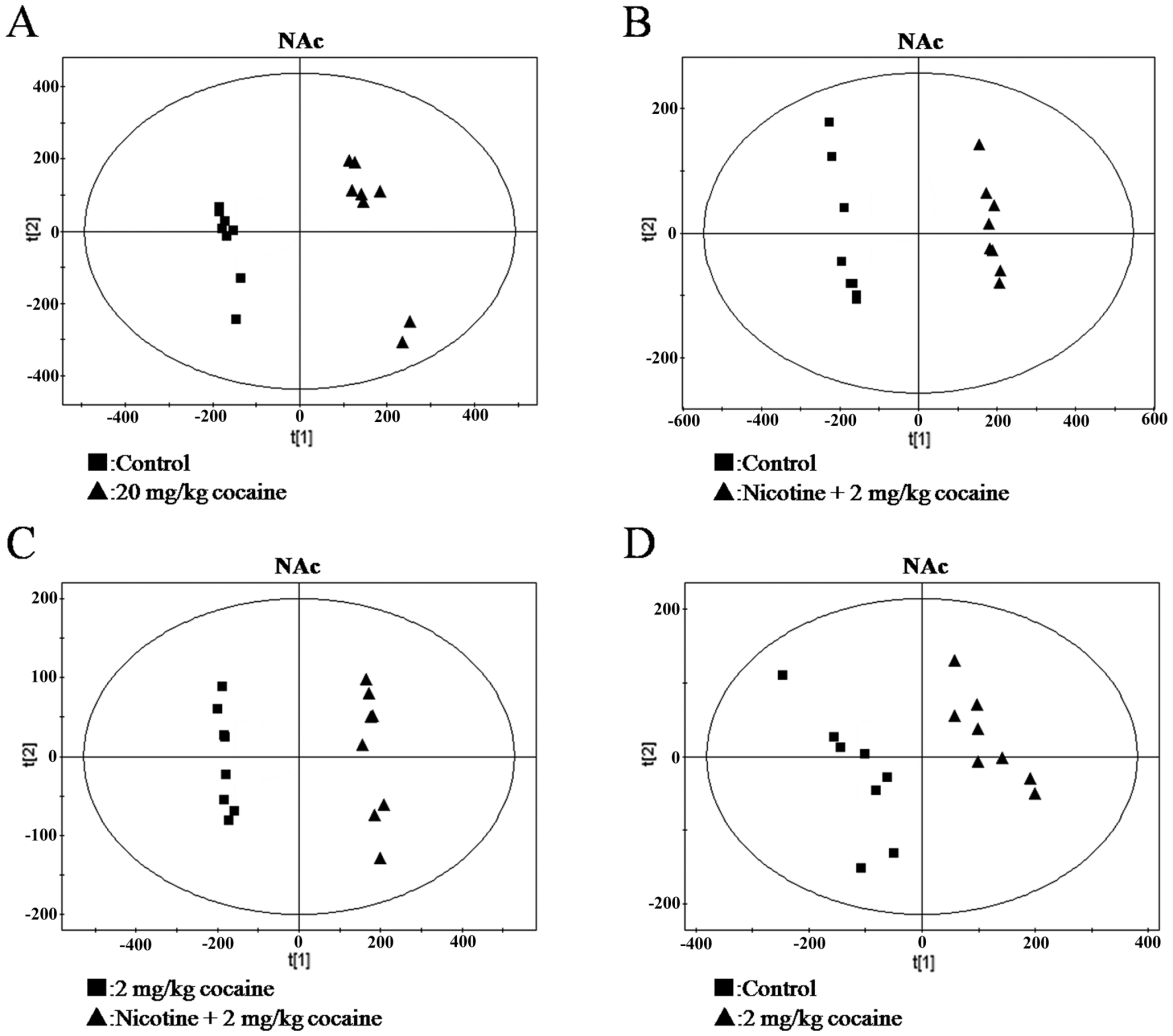
OSC-PLS scores plots of NAc from mice. (A) control vs. 20 mg/kg cocaine; (B) control vs. nicotine +2 mg/kg cocaine; (C) 2 mg/kg cocaine vs. nicotine +2 mg/kg cocaine; (D) control vs. 2 mg/kg cocaine.

**Figure 4:**
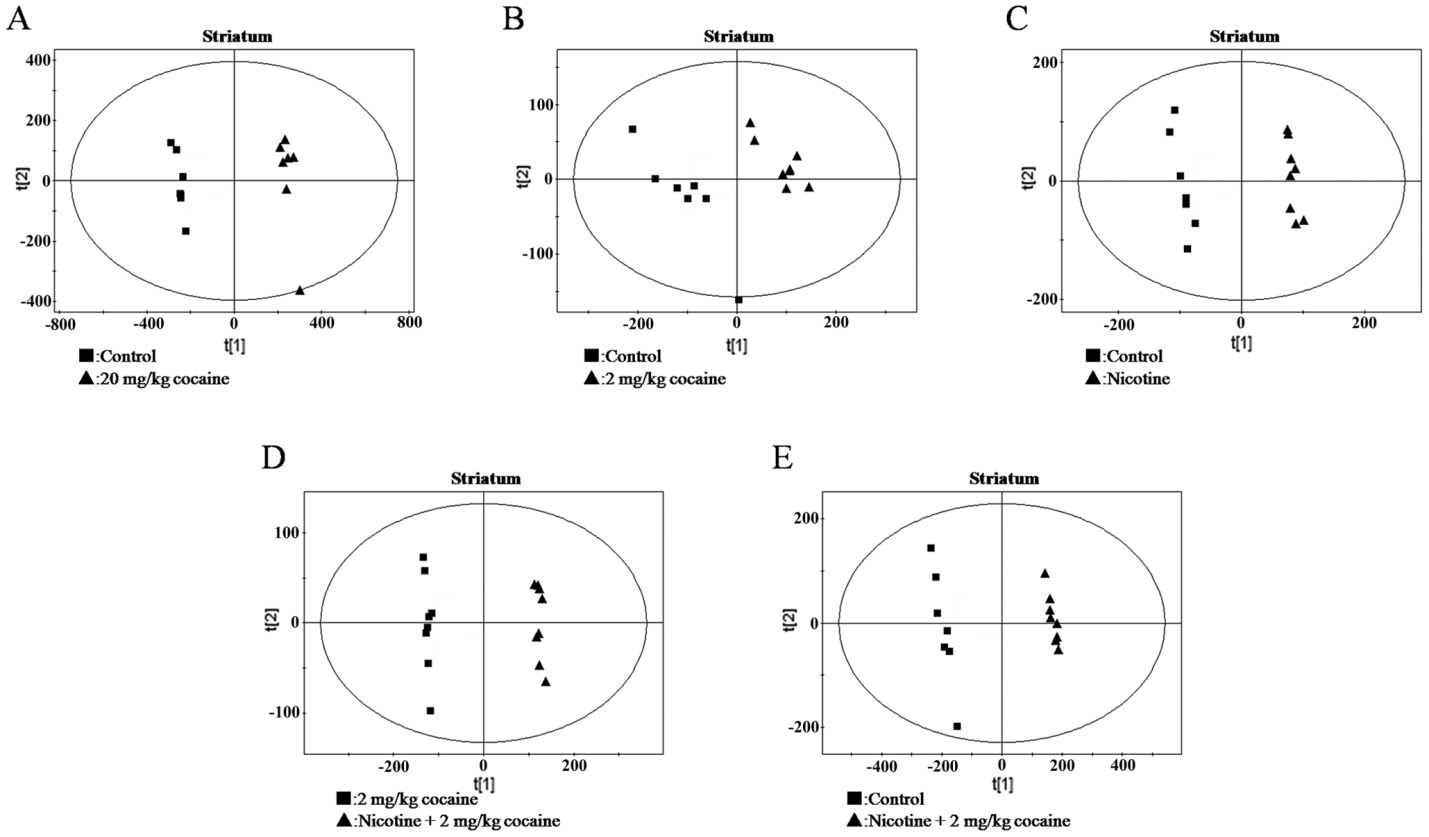
OSC-PLS scores plots of striatum from mice. (A) control vs. 20 mg/kg cocaine; (B) control vs. nicotine +2 mg/kg cocaine; (C) 2 mg/kg cocaine vs. nicotine +2 mg/kg cocaine; (D) control vs. nicotine; (E) control vs. 2 mg/kg cocaine.

Representative ^1^H NMR spectra of the water extracts of striatum from five groups were shown and major metabolites in the integrate regions were assigned in Figure 2. Visual inspection of ^1^H-NMR spectra indicated the differences in these groups. We further utilized PCA, PLS and OSC to gain insights into biochemical information at the molecular level from ^1^H NMR spectra. As an unsupervised PR method, PCA was initially used to analyze the data sets of the ^1^H NMR spectra. Subsequently, PLS, a supervised PR method, was applied to increase this separation. However, there was no clear separation in the brain NMR spectra for the first two principal components (PCs) in each treated group when these two PR methods were used. Then, PLS model following OSC was performed to separate NMR spectra among treated groups. After application of OSC-PLS model, the PLS scores plots displayed a significant differentiation among treated groups (Figure 3. and Figure 4.).

**Figure 5:**
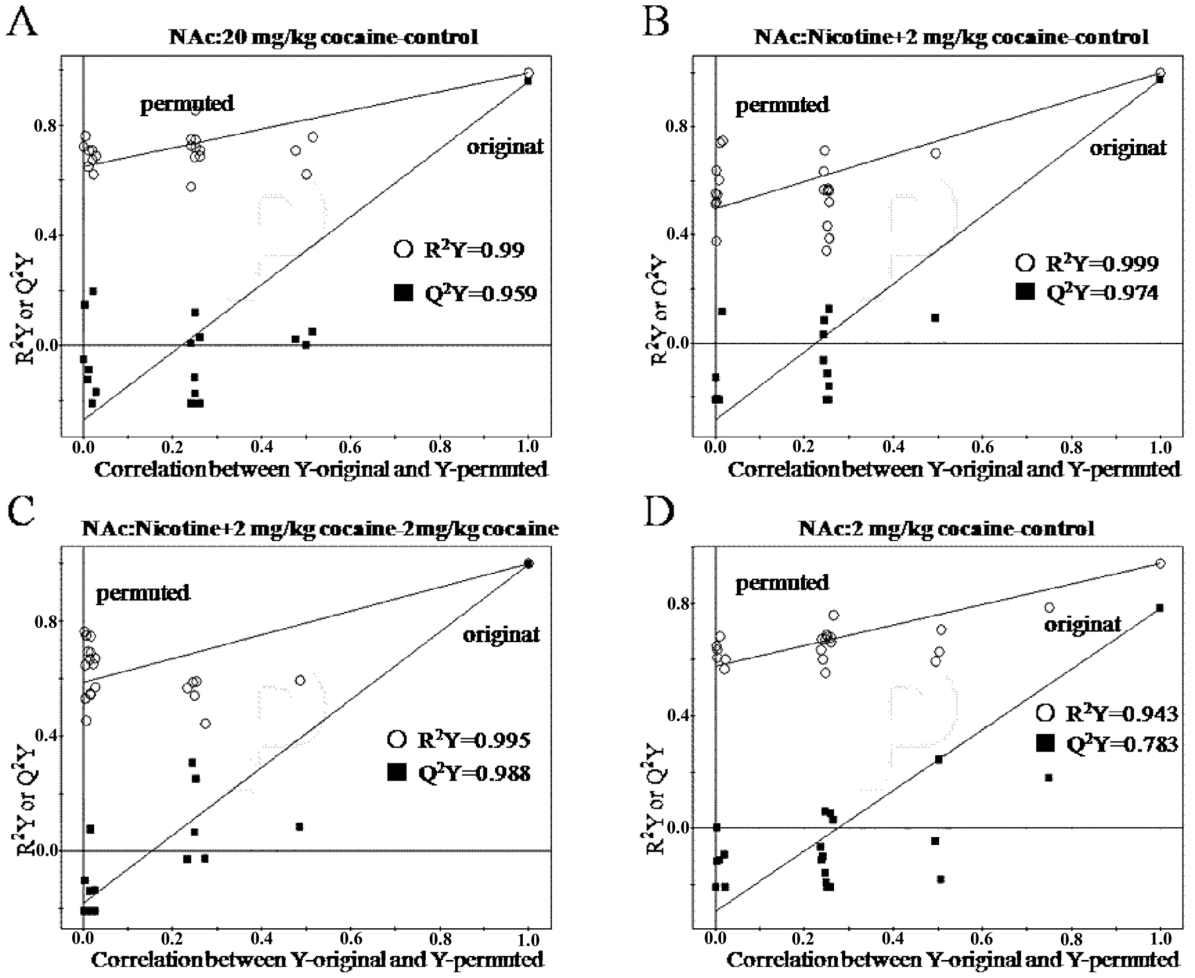
Validation plots of the PLS models after application of OSC in NAc. (A) control vs. 20 mg/kg cocaine; (B) control vs. nicotine +2 mg/kg cocaine; (C) 2 mg/kg cocaine vs. nicotine +2 mg/kg cocaine; (D) control vs. 2 mg/kg cocaine.

**Figure 6:**
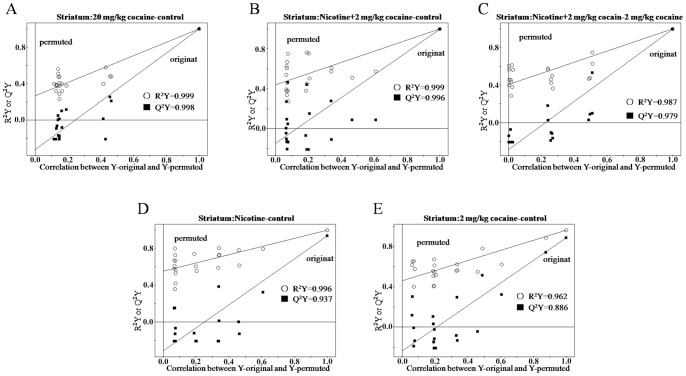
Validation plots of the PLS models after application of OSC in striatum. (A) control vs. 20 mg/kg cocaine; (B) control vs. nicotine +2 mg/kg cocaine; (C) 2 mg/kg cocaine vs. nicotine +2 mg/kg cocaine; (D) control vs. nicotine; (E) control vs. 2 mg/kg cocaine.

In order to confirm the quality and effectiveness of OSC-PLS model, a permutation method was used. The original data points (0∼1) in the training set were mathematically reproduced, and 1 indicates a model with a perfect fit. Q^2^Y values >0.5 and >0.9 express good and excellent predictive abilities, respectively. High values of R^2^Y and Q^2^Y in PLS models in our study indicated that OSC-PLS model was valid (Figure 5. and Figure 6.). For example, R^2^Y and Q^2^Y values of NAc samples from 20 mg/kg cocaine group were 0.99 and 0.959, respectively (Figure 5.).

### 4. Metabolic Changes Induced by Drugs in Brain NAc and Striatum of Mice

**Figure 7:**
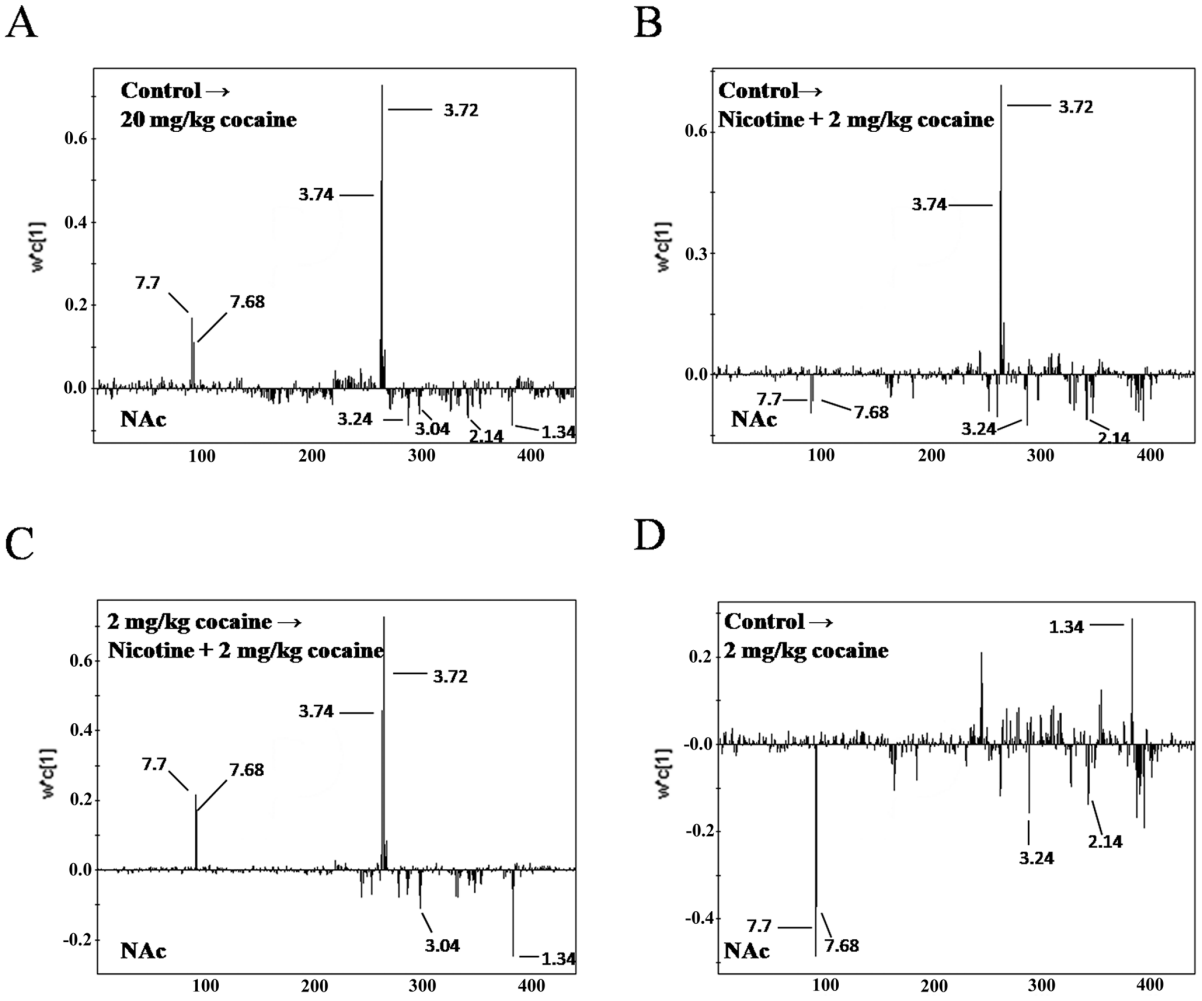
Comparison of NAc from mice by applying PLS loading plots for the region 9.4–5.1 and 4.6-0.2 ppm (440 segments) after application of OSC data filter. (A) control vs. 20 mg/kg cocaine; (B) control vs. nicotine +2 mg/kg cocaine; (C) 2 mg/kg cocaine vs. nicotine +2 mg/kg cocaine;(D) control vs. 2 mg/kg cocaine.

**Figure 8:**
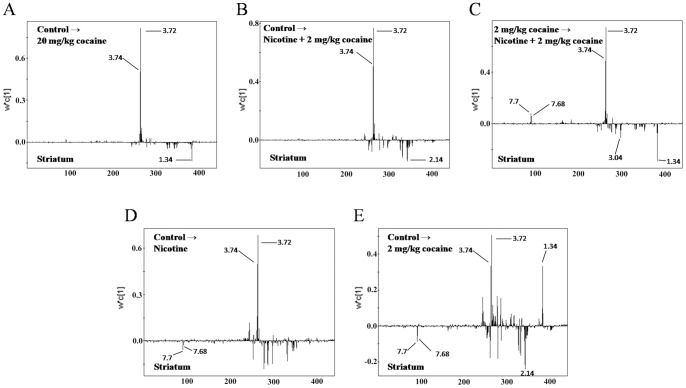
Comparison of striatum from mice by applying PLS loading plots for the region 9.4–5.1 and 4.6-0.2 ppm (440 segments) after application of OSC data filter. (A) control vs. 20 mg/kg cocaine; (B) control vs. nicotine +2 mg/kg cocaine; (C) 2 mg/kg cocaine vs. nicotine +2 mg/kg cocaine; (D) control vs. nicotine; (E) control vs. 2 mg/kg cocaine.

To investigate the changes of metabolites in NAc and striatum, PLS loading plots after application of OSC data filter were applied to analyze ^1^H NMR data (Figure 7. and Figure 8.). In NAc, the metabolites that predominantly contributed to the separation of drug-treated groups were glutamate (Glu), tryptamine, glucose, lactate (Lac), creatine (Cre), 1-methylhistidine, glutamine (Gln) and profine (Figure 7. and Table 2). In striatum, the metabolites that predominantly contributed to the separation were Glu, glucose, a-ketogultaric acid, Lac and Gln (Figure 8. and Table 3). Interestingly, most of these modified metabolites in 20 mg/kg cocaine group (high-dose model) were overlapped with those in combinational drugs group (threshold dose model), revealing an analogous outcome at metabolic profiling (Table 2 and Table 3). Several identical metabolites modified in these two models were also observed in 2 mg/kg cocaine or nicotine alone groups. However, a few metabolites were not significantly changed in 2 mg/kg cocaine group, but were modified clearly when pretreated with nicotine, suggesting that these metabolites were supplied by nicotine priming (Table 2 and Table 3). Correspondingly, according to the VIP values (VIP≥1) in OSC-PLS model, these changed metabolites in drug groups were identified and listed in Table 2 and Table 3, respectively.

**Table 2:** Summary of the variations from NAc metabolites in mice.

Metabolites^a^	Chemicalshift (ppm)	Control vs. 20 mg/kgcocaine		Control vs. nicotine+2 mg/kg cocaine		2 mg/kg cocaine vs.nicotine +2 mg/kg cocaine		Control vs.2 mg/kg cocaine	
		VIP^b^	Loading^c^	VIP^b^	Loading^c^	VIP^b^	Loading^c^	VIP^b^	Loading^c^
Glutamate	3.72	14.95	+0.728	14.91	+0.715	15.19	+0.726	1.16	+0.057
Glutamate	3.74	10.23	+0.498	9.45	+0.453	9.57	+0.457	/	/
Tryptamine	7.70	4.54	+0.171	2.05	−0.098	4.52	+0.216	9.55	−0.487
Tryptamine	7.68	2.99	+0.112	1.43	−0.068	3.64	+0.174	7.34	−0.374
Glucose	3.76	2.41	+0.117	/	/	/	/	1.99	−0.102
Lactate	1.34	2.05	−0.089	1.23	−0.059	5.26	−0.249	6.48	+0.288
*	3.66	1.92	+0.094	2.66	+0.128	1.73	+0.083	1.84	+0.081
Acetylcholine	3.24	1.83	−0.089	/	/	/	/	3.32	−0.159
1-Methylhistidine	3.70	1.57	+0.076	1.51	+0.073	1.52	+0.072	1.02	+0.014
L-glutamine	2.14	1.45	−0.071	2.37	−0.113	/	/	2.28	−0.114
L-methionine	2.16	1.37	−0.065	2.38	−0.114	/	/	2.75	−0.139
Creatine	3.04	1.27	−0.062	1.38	−0.065	2.37	−0.113	1.29	+0.066
α-Ketogultaric acid	2.48	1.14	−0.055	1.47	−0.071	/	/	1.96	−0.09
1-Methylhistidine	3.68	1.08	+0.052	/	/	/	/	/	/
α-Ketogultaric acid	2.46	1.07	−0.051	1.53	−0.073	/	/	2.05	−0.099
*	3.56	1.06	−0.051	/	/	/	/	1.01	+0.012
Phosphocholine	3.22	1.03	−0.048	/	/	/	/	1.22	+0.049
Proline	4.12	1.01	+0.047	1.24	+0.058	1.68	−0.08	4.15	+0.211

a Metabolites: (*) represents a material which has not been identified.

b Variable importance in the projection (VIP≥1) was obtained from OSC-PLS. Oblique line (/) represents no variations of the metabolite between the left group of the subtitle and the right group of the subtitle.

c Loading: the positive (+) value represents an increase of the metabolite in the right group of the subtitle (e.g.: 20 mg/kg cocaine group of control vs. 20 mg/kg cocaine); while the negative (−) value represents a decrease of the metabolite in the right group of the subtitle; Oblique line (/) represents no variations of the metabolite between the left group of the subtitle and the right group of the subtitle.

**Table 3:** Summary of the variations from striatum metabolites in mice.

Metabolites^a^	Chemicalshift (ppm)	Control vs.20 mg/kg cocaine		Control vs.Nicotine +2 mg/kg cocaine		2 mg/kg cocaine vs.nicotine +2 mg/kgcocaine		Control vs.nicotine		Control vs.2 mg/kg cocaine	
		VIP^b^	Loading^c^	VIP^b^	Loading^c^	VIP^b^	Loading^c^	VIP^b^	Loading^c^	VIP^b^	Loading^c^
Glutamate	3.72	17.04	+0.816	15.98	+0.767	15.61	+0.746	14.28	+0.684	9.76	+0.506
Glutamate	3.74	10.52	+0.504	10.49	+0.503	10.20	+0.487	10.35	+0.496	6.51	+0.335
Lactate	1.34	2.92	−0.138	/	/	6.12	−0.291	1.26	−0.024	7.06	+0.332
*	3.66	2.12	+0.102	2.35	+0.113	1.62	+0.076	1.69	+0.081	2.23	+0.116
1-Methylhistidine	3.70	1.62	+0.077	/	/	/	/	/	/	/	/
Glucose	3.76	1.24	+0.059	/	/	2.05	+0.098	3.34	+0.159	1.67	−0.084
1-Methylhistidine	3.68	1.21	+0.058	/	/	/	/	/	/	/	/
α-Ketogultaric acid	2.46	1.08	−0.051	1.62	−0.074	/	/	/	/	2.87	−0.139
Lactate	1.36	1.03	−0.049	/	/	1.53	−0.073	/	/	1.80	+0.095
L-glutamine	2.14	1.00	−0.048	3.08	−0.146	/	/	/	/	4.60	−0.242

a Metabolites: (*) represents a material which has not been identified.

b Variable importance in the projection (VIP≥1) was obtained from OSC-PLS. Oblique line (/) represents no variations of the metabolite between the left group of the subtitle and the right group of the subtitle.

c Loading: the positive (+) value represents an increase of the metabolite in the right group of the subtitle (e.g.: 20 mg/kg cocaine group of control vs. 20 mg/kg cocaine); the negative (−) value represents a decrease of the metabolite in the right group of the subtitle; Oblique line (/) represents no variations of the metabolite between the left group of the subtitle and the right group of the subtitle.

### 5. Neurotransmitter Changes

We found that Glu, tryptamine, Gln and 1-methylhistidine were markedly altered in NAc and striatum after treatment of nicotine or cocaine. Glu (3.72 and 3.74 ppm) in NAc and striatum was increased by 2 mg/kg cocaine. With nicotine pretreatment, 2 mg/kg cocaine significantly increased the level of Glu. Interestingly, Glu was also significantly elevated by 20 mg/kg cocaine (Table 2 and Table 3). Markedly changed neurotransmitters in NAc and striatum are listed in Table 2 and Table 3.

We then carefully analyzed the altered metabolites among treatment groups. We found that tryptamine (7.7 and 7.68 ppm) clearly decreased in 2 mg/kg cocaine group, but only slightly reduced in nicotine +2 mg/kg cocaine group. Moreover, tryptamine was slightly increased by 20 mg/kg cocaine (Table 4). Additionally, Gln in NAc and striatum were markedly lowered by 2 mg/kg cocaine alone, but only showed a little decrease with nicotine preconditioning. Interestingly, a little decrease of Gln also displayed in 20 mg/kg cocaine alone group (Table 4 and Table 5). These results showed that after nicotine pretreatment, neurotransmitter profiles induced by subsequent 2 mg/kg cocaine shifted toward that of 20 mg/kg cocaine group.

**Table 4:** Contrast of the variations from NAc metabolites in different groups in mice.

Metabolites^a^	Chemicalshift (ppm)	Control vs.20 mg/kg cocaine	Control vs.nicotine +2 mg/kg cocaine	2 mg/kg cocaine vs.nicotine +2 mg/kg cocaine	Control vs.2 mg/kg cocaine
		Loading^b^	Loading^b^	Loading^b^	Loading^b^
Glutamate	3.72	++	++	++	+
Glutamate	3.74	++	++	++	/
Tryptamine	7.70	++	−	+	–
Tryptamine	7.68	++	−	+	–
Glucose	3.76	+	/	/	−
Lactate	1.34	–	−	−	++
*	3.66	+	++	++	+
Acetylcholine	3.24	−	/	/	−
1-Methylhistidine	3.70	++	++	++	+
L-glutamine	2.14	−	−	/	−
L-methionine	2.16	−	−	/	−
Creatine	3.04	−	−	−	+
α-Ketogultaric acid	2.48	−	−	/	−
1-Methylhistidine	3.68	+	/	/	/
α-Ketogultaric acid	2.46	−	−	/	−
*	3.56	−	/	/	+
Phosphocholine	3.22	−	/	/	+
Proline	4.12	+	+	−	++

a Metabolites: (*) represents a material which has not been identified.

b Loading: (+) represents a somewhat increase of the metabolite in the right group of the subtitle(e.g.: 20 mg/kg cocaine group of control vs. 20 mg/kg cocaine); (++) represents a significant increase of the metabolite in the right group of the subtitle; (−) represents a decrease of the metabolite in the right group of the subtitle; (–) represents a significant decrease of the metabolite in the right group of the subtitle; (/) represents no variations of the metabolite between the left group of the subtitle and the right group of the subtitle.

**Table 5:** Contrast of the variations from striatum metabolites in different groups in mice.

Metabolites^a^	Chemical shift(ppm)	Control vs.20 mg/kg cocaine	Control vs. nicotine+2 mg/kg cocaine	2 mg/kg cocaine vs.nicotine +2 mg/kg cocaine	Control vs.Nicotine	Control vs.2 mg/kg cocaine
		Loading^b^	Loading^b^	Loading^b^	Loading^b^	Loading^b^
Glutamate	3.72	++	++	+	++	+
Glutamate	3.74	++	++	+	++	+
Lactate	1.34	−	/	−	–	+
*	3.66	++	++	+	++	+
1-Methylhistidine	3.70	+	/	/	/	/
Glucose	3.76	+	/	+	++	−
1-Methylhistidine	3.68	+	/	/	/	/
α-Ketogultaric acid	2.46	−	−	/	/	−
Lactate	1.36	−	/	−	/	+
L-glutamine	2.14	−	−	/	/	−

a Metabolites: (*) represents a material which has not been identified.

b Loading: (+) represents a somewhat increase of the metabolite in the right group of the subtitle (e.g.: 20 mg/kg cocaine group of control vs. 20 mg/kg cocaine); (++) represents a significant increase of the metabolite in the right group of the subtitle; (−) represents a decrease of the metabolite in the right group of the subtitle; (–) represents a significant decrease of the metabolite in the right group of the subtitle; (/) represents no variations of the metabolite between the left group of the subtitle and the right group of the subtitle.

### 6. Modification in Energy Metabolism

As listed in Table 2 and Table 3, several metabolites related to energy metabolism including glucose, Lac, creatine and α-ketoglutaric acid, were markedly modified by nicotine or cocaine. Compared with the control group, glucose (3.76 ppm) in striatum was significantly decreased by 2 mg/kg cocaine; however, it showed no obvious difference in combinational drugs group. Glucose exhibited a slight increase in 20 mg/kg cocaine group (Table 4 and Table 5). It is known that Cre stores energy for the cell by means of a phosphate covalent bond in a similar manner to ATP/ADP. Interestingly, Cre (3.04 ppm) in NAc showed a little high level in 2 mg/kg cocaine group. However, nicotine pretreatment followed by 2 mg/kg cocaine led to a decreased Cre level in NAc, which was also observed in 20 mg/kg cocaine group. Lac is an alternate energy source for brain. We found that Lac (1.34 ppm) in drug groups showed similar change to Cre. As compared with the control, α-ketoglutaric acid (2.46 and 2.48 ppm) in NAc and striatum displayed a little decline after nicotine pretreatment, and was further decreased by following treatment of 2 mg/kg cocaine. Interestingly, a decline in α-ketoglutaric acid was also observed in high-dose model of cocaine (Table 5). These results indicate that nicotine pretreatment can modify the profiles of energy substances of 2 mg/kg cocaine more akin to that of 20 mg/kg cocaine. Meanwhile, these findings suggest that nicotine may create a susceptible environment to conditioned rewarding effects of cocaine, at least in part, through modifying some specific energy substances.

### 7. Disruption of Membrane and Amino Acids

Phosphocholine (3.22 ppm), a membrane ingredient, was elevated by 2 mg/kg cocaine alone; however, it showed no obvious alteration in nicotine +2 mg/kg cocaine group or nicotine alone group. A somewhat decline in phosphocholine was induced by 20 mg/kg cocaine (Table 2). Proline (4.12 ppm) in NAc displayed a slightly low level after nicotine treatment, whereas it was markedly elevated by 2 mg/kg cocaine alone (Table 2). Interestingly, increased proline level in threshold dose model was also found in high-dose model. Additionally, L-methionine (2.16 ppm) was slightly declined by nicotine pretreatment, whereas it was significantly decreased by following 2 mg/kg cocaine. Surprisingly, a decline of L-methionine in threshold model was also observed in high-dose model (Table 4 and Table 5). These results indicate that after nicotine pretreatment, reprogramming of metabolites induced by a low dose of cocaine (2 mg/kg cocaine) is extremely comparable with that induced by a high dose of cocaine (20 mg/kg cocaine) alone.

### 8. Discovery of Key Metabolites Related to Rewarding Effects of Cocaine Elicited by Nicotine

**Figure 9:**
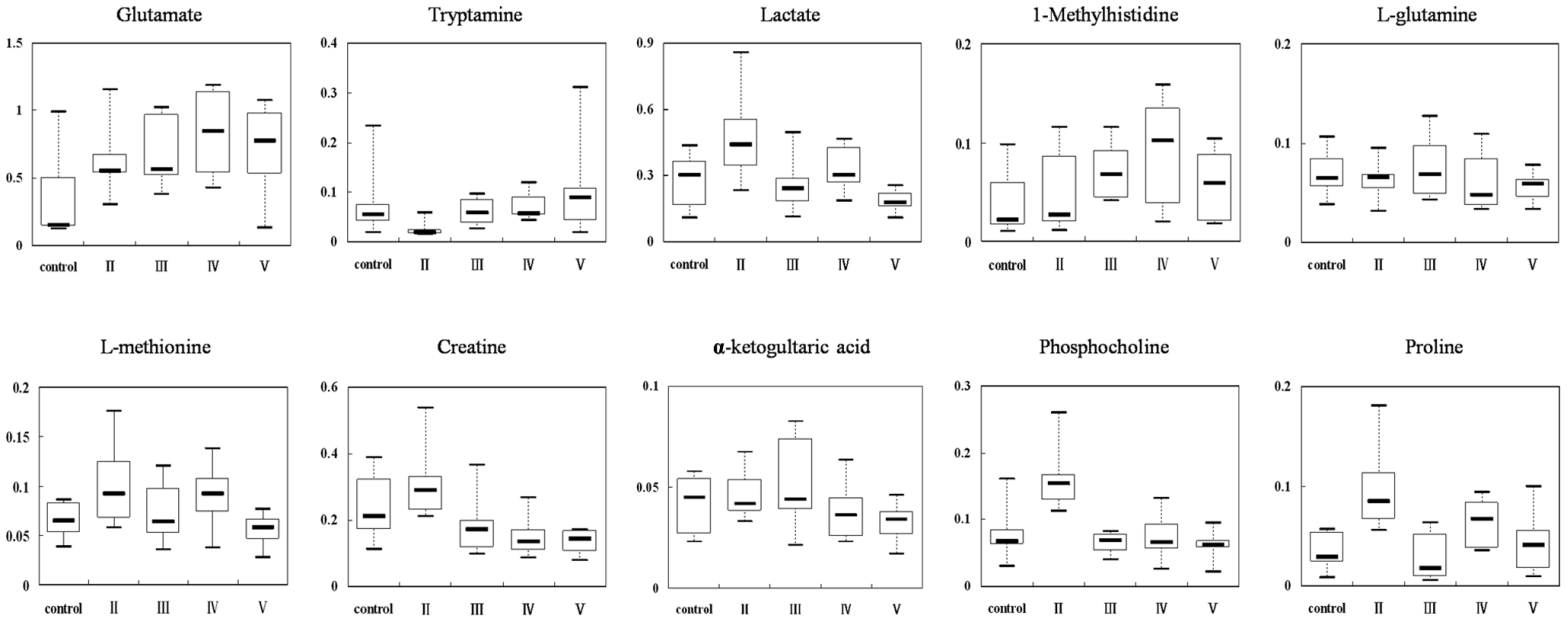
Box-and-whisker plots illustrate progressive changes of the metabolites among 5 treatment groups: saline (control), 2 mg/kg cocaine (II), nicotine (III), nicotine +2 mg/kg cocaine (IV) and 20 mg/kg cocaine (V). Horizontal line in the middle portion of the box, median; bottom and top boundaries of boxes, lower and upper quartile; whiskers, 5th and 95th percentiles; open circles, outliers.

**Figure 10:**
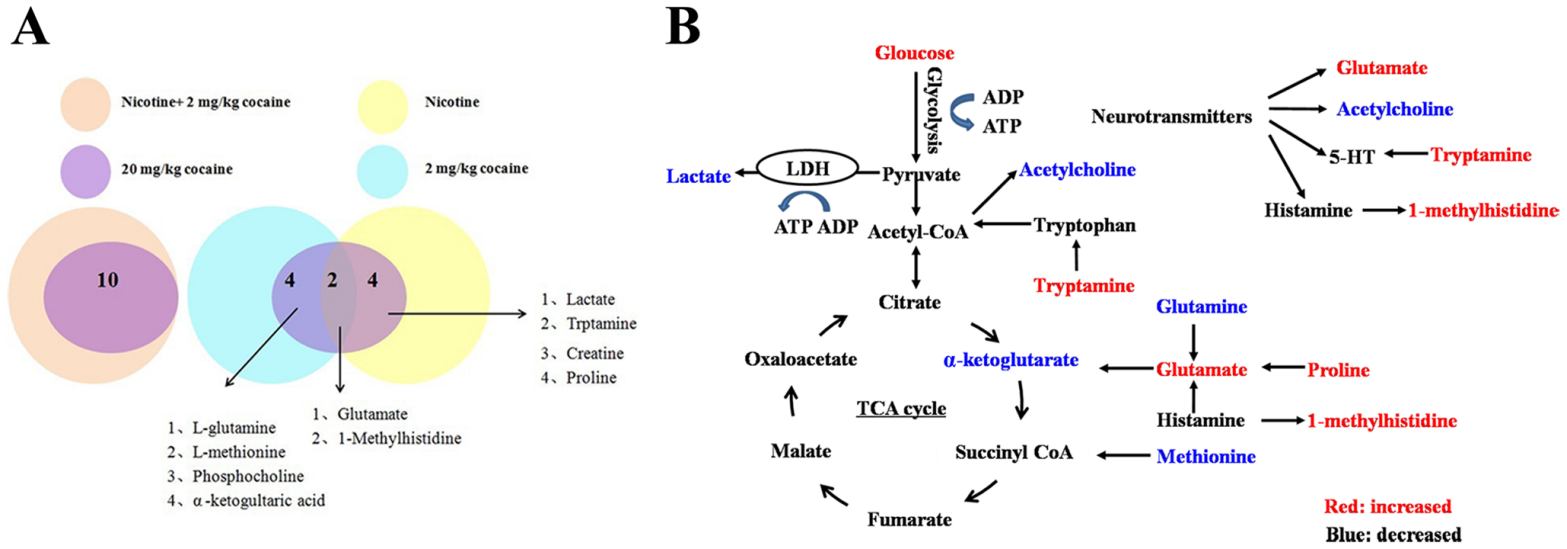
Changed metabolites in brain NAc and altered metabolic pathways for the most relevant distinguishing metabolites with nicotine or cocaine treatment. (A) In the left, changed metabolites in 20 mg/kg cocaine group exhibit similar changes with combinational drugs group, sharing 10 overlapped metabolites. In the right, a part of modified metabolites showed considerable overlap between 20 mg/kg cocaine group and 2 mg/kg cocaine group or nicotine group. Among them, two shared metabolites between 2 mg/kg cocaine group and nicotine group are Glu and 1-methylhistidine; four shared metabolites between 2 mg/kg cocaine group and 20 mg/kg cocaine group are Gln, L-methionine, α-ketoglutaric acid and phosphocholine; four shared metabolites between nicotine group and 20 mg/kg cocaine group are tryptamine, Lac, Cre and proline. (B) Blue metabolites are upregulated, and red metabolites are downregulated.

To further gain an insight into the key metabolites associated with rewarding effects of cocaine primed by nicotine pretreatment, we used box-and-whisker plots to clarify altered levels of those identified metabolites. We obtained 12 representative metabolites among these five treatment groups (saline, 2 mg/kg cocaine, nicotine, nicotine +2 mg/kg cocaine and 20 mg/kg cocaine). Interestingly, 10 identical metabolites in NAc showed a similar change between 20 mg/kg cocaine group and nicotine +2 mg/kg cocaine group, including Glu, tryptamine, Lac, 1-methylhistidine, Gln, L-methionine, Cre, α-ketoglutaric acid, phosphocholine and proline (Figure 9.). Some modified metabolites in above two models were overlapped with those in 2 mg/kg cocaine group or nicotine group, such as Glu and 1-methylhistidine. Four metabolites, Gln, L-methionine, α-ketoglutaric acid and phosphocholine, showed a consistent trend only in 2 mg/kg cocaine group. Tryptamine, Lac, Cre and proline were altered only in nicotine group (Figure 10A.). The change tendency of above metabolites in NAc was also observed in striatum. These results show that with nicotine pretreatment threshold dose of cocaine could reprogram and shift the metabolic profiles in NAc and striatum to that of high dose cocaine. These modified metabolites mainly included neurotransmitter, energy substances, membrane components and amino acids. For example, Cre in NAc showed somewhat high level in 2 mg/kg cocaine group; however, after nicotine pretreatment, 2 mg/kg cocaine clearly decreased its level to that of 20 mg/kg cocaine group. Meanwhile, Lac displayed a similar alteration to Cre. The α-ketoglutaric acid in NAc and striatum was significantly decreased by 2 mg/kg cocaine, whereas it displayed a slight decline in combinational drugs group, which was consistent with the findings in 20 mg/kg cocaine group (Table 5). Taken above, after nicotine pretreatment, metabolic profiling of threshold dose of cocaine shifts to that of high-dose of cocaine. We consider that nicotine priming may provide a favorable metabolite environment in brain, facilitating the rewarding effect of subsequent cocaine.

## Discussion

Addictive drugs, including heroin, marijuana, nicotine and cocaine, exert their addictive effects in part by increasing the level of dopamine, a pivotal neurotransmission in the control of initial drug use in the ventral striatum [24], [25]. Nicotine and cocaine have interactive neurochemical effects, particularly with regard to dopamine transmission [26]. Many studies have been performed to explore the effect of nicotine on cocaine. For example, nicotine exposure in adolescence has been shown to alter the rewarding effect of cocaine in adulthood [27]. Nicotine enhances locomotor activity to cocaine and activates other indirect dopamine agonists [28], [29]. However, these studies mainly focus on certain genes or neurotransmitters, and cannot understand the underlying mechanism from systems biology framework. It is well known that NAc and striatum are the two key components of brain’s reward circuitry [30], [31]. Therefore, we applied ^1^H NMR-based on metabonomic in NAc and striatum to study the effect of nicotine preconditioning on cocaine.

With nicotine pretreatment,low dose of cocaine (2 mg/kg) dramatically increased CPP of mice. Interestingly, the mice receiving 20 mg/kg cocaine or nicotine +2 mg/kg cocaine displayed not only analogous CPP but also extremely similar metabolic profiling. In other words, nicotine pretreatment moved the metabolic profiling of threshold dose of cocaine to that of high dose. We consider that the effect of nicotine on reinforcing behavioral response to cocaine may be attributed to nicotine-induced modification of some specific metabolites, thus creating a favorable environment of metabolites for conditioned rewarding effects of cocaine. The changes of metabolites in NAc and striatum involved in neurotransmitter disturbance, energy metabolism imbalance, membrane and amino acids disruptions.

### 1. Disturbance in Neurotransmitters Specific to Nicotine and Cocaine Treatment

Interactions between nicotine and cocaine have been studied in rodents, and it has been found that nicotine can enhance cocaine’s behavioral effects [28]. Drug-related learning, memory and sensitization are linked to a glutamate-mediated LTP induction and maintenance at these synapses of smokers [32], [33], [34]. In this study, Glu in NAc and striatum was slightly increased by 2 mg/kg cocaine alone. Surprisingly, after nicotine pretreatment, Glu was dramatically increased by low-dose cocaine (2 mg/kg), reaching to the level of high dose (20 mg/kg). Gln in NAc was decreased similarly in threshold dose and high-dose model of cocaine. We guess that the reduction in Gln could be attributed to being hydrolyzed to Glu. Previous studies have shown that cocaine indirectly influences Glu transmission in the limbic system, producing persistent changes in neuronal function that alters behavioral effect of cocaine [35], [36], [37]. Similarly, reinstatement of cocaine seeking is linked to the increased Glu release in NAc [38]. Thinking above, in the present study, increase of Glu induced by nicotine should contribute to the induction of glutamatergic synaptic plasticity, likely through activation of N-methyl-D-aspartic acid receptor (NMDAR) [39]. Glu increases glutamate release onto DA neurons in ventral tegmental area, and initially activates NMDA receptor-mediated signaling cascades, which, in turn, upregulate α-amino-3-hydroxy-5-methyl-4-isoxazole-propionic acid receptors and induce glutamatergic synaptic plasticity [40]. We speculate that nicotine’s reinforcing effect on cocaine CPP, at least in part, owe to the increase of Glu release, thus leading to Glu receptor activation and modification of glutamatergic synaptic plasticity.

Additionally, we found that nicotine pretreatment increased tryptamine level in NAc, which was further elevated by subsequent 2 mg/kg cocaine. Interestingly, the elevated level of tryptamine in threshold dose model was similar to that in high-dose model. It is known that 5-HT, a derivative of tryptamine, plays a role in initiation and maintenance of addictive behavior [41]. Therefore, elevated tryptamine may play a part in the reinforcing effect of nicotine on cocaine. Collectively, our results, together with previous studies, suggest that nicotine exerts its effect on cocaine though creating a specific environment of metabolite, thus reinforcing conditioned rewarding effects of cocaine.

### 2. Dysregulation in Energy Metabolism Related to Nicotine and Cocaine Treatment

Brain energy supply almost entirely requires oxidative metabolism of glucose in mitochondria and demands lactic acid from glycolytic processes [42]. Neuronal activity is extremely energy demanding [43]. In our study, several metabolites related to energy metabolism, such as glucose, Lac, creatine, glycine and α-ketoglutaric acid, were markedly modified by nicotine or combinational drugs. Importantly, changes of these metabolites were analogous. Glucose, a primary energy substrate for brain metabolism [44], plays an important role in energy homeostasis [45], [46]. We found that glucose in striatum was reduced by 2 mg/kg cocaine, but it showed no obvious difference in nicotine +2 mg/kg cocaine group. Glucose in striatum showed a slight increase in high-dose group. These results indicate that with nicotine priming glucose level in threshold dose group shifted toward that in high-dose group.

Chronic administration of nicotine causes a significant increase in glucose transport protein densities and local cerebral glucose utilization [47]. Additionally, nicotine is capable of effectively improving learning and cognitive functions in various species [48], [49]. It is reported that cognition is highly energy dependent and that glucose is used by neurons as main energy substrate [50]. Therefore, nicotine-induced glucose increase in the present study may reflex a stressful increase of energy supply in brain. Moreover, Cre stores energy for the cell by means of a phosphate covalent bond in a similar manner to ATP/ADP [51]. Cre has recently been implicated in energy homeostasis and direct antioxidant effects [18], [52]. We found that Cre in NAc was somewhat elevated by 2 mg/kg cocaine. However, after nicotine pretreatment, 2 mg/kg cocaine decreased the level of Cre. Interestingly, low level of Cre was observed in both threshold dose but also high-dose model, indicating that rewarding effects of addictive drugs need to consume a lot of energy. Lac is deemed to supply an alternate energy sources for brain and could be associated with protective preconditioning [42]. Meanwhile, Lac is metabolized through the tricarboxylic acid cycle (TCA) and when compared to glucose, it is equivalent with regard to its access to the TCA in neuron [53]. We found that Lac in drug-treated groups also showed similar change to Cre. Additionally, α-ketoglutaric acid is produced to supply energy as a substrate of TCA. Our results showed that nicotine decreased the levels of Cre, Lac and α-ketoglutaric in NAc and striatum; moreover, the levels of these metabolites were comparable between threshold dose and high-dose model. We guess that the metabolites modified by nicotine may reflect an energetic shift between different brain regions and alteration of energy storage capacity, favoring subsequent reinforcing effect of cocaine. Taken above, it is reasonable to infer that nicotine priming provides a beneficial energy supply for conditioned rewarding effect of cocaine.

### 3. Membrane Disruption and Amino Acids Related to Metabolites Change

Phosphocholine, a precursor to membrane phospholipids in the cell, is the major choline phospholipid metabolites [51]. Membrane damage results in release of phospholipids and choline compounds, the major head of phospholipids [54]. In this study, phosphocholine in NAc was significantly increased by 2 mg/kg cocaine, but it showed no alteration when the mice was pretreated with nicotine. A slight decline in phosphocholine was induced by 20 mg/kg cocaine. Change of phosphocholine may be the potential indication of cell membrane disruption in transportation or barrier function.

Disorder of amino acid metabolism is probably induced by proteolysis, oxidative catabolism, and gluconeogenesis [55], [56]. Amino acids as substrates are highly demanded for energy production during infection [57]. Nicotine can affect transport processes of different classes of amino acids in human placental villus [58]. Several studies have showed that a subset of six metabolites including proline, sarcosine, uracil, kynurenine, glycerol-3-phosphate and leucine, significantly increase in metastatic prostate cancer and may be regarded as biomarkers for progressive disease [59]. We showed that proline, L-methionine and Glu were significantly altered by nicotine or cocaine. Because these amino acids belong to essential amino acids, non-essential amino acids or amino acid with putative neurotransmitter function, our findings suggest that nicotine pretreatment may influence protein metabolism through modifying amino acids in brain.

In summary, this study applied ^1^H NMR-based metabonomics in two brain regions to explore the mechanism by which nicotine increases behavioral response to cocaine. Our results show that nicotine priming can supply a beneficial environment of metabolites for reinforcing rewarding effects of cocaine. The modified metabolites include neurotransmitter, energy source and amino acids, which may be the outcome of the adaptive measures taken by the brain in response to nicotine priming. The related metabolic pathways are summarized in Figure 10B. These results from animals prompt an analysis of epidemiological data, which display that most cocaine users have smoked cigarettes before they began to use cocaine and that dependence of cocaine after smoking is further increased. Moreover, our findings suggest that effective interventions to smoking would not only prevent its negative health consequences but could also decrease the risk of progression to chronic illicit drug use.

## References

[1] Kandel D (1975) Stages in adolescent involvement in drug use. Science 190: 912–914. 1188374 10.1126/science.1188374

[2] Torabi MR, Bailey WJ, Majd-Jabbari M (1993) Cigarette smoking as a predictor of alcohol and other drug use by children and adolescents: evidence of the “gateway drug effect”. Journal of School Health 63: 302–306. 8246462 10.1111/j.1746-1561.1993.tb06150.x

[3] O’Donnell JA (1979) Cigarette smoking as a precursor of illicit drug use. Cigarette Smoking as a Dependence Process: 30. 111138

[4] Levine A, Huang Y, Drisaldi B, Griffin EA Jr, Pollak DD, et al (2011) Molecular mechanism for a gateway drug: epigenetic changes initiated by nicotine prime gene expression by cocaine. Science translational medicine 3: 107–109. 10.1126/scitranslmed.3003062 PMC4042673 22049069

[5] Kelley BM, Rowan JD (2004) Long-term, low-level adolescent nicotine exposure produces dose-dependent changes in cocaine sensitivity and reward in adult mice. International journal of developmental neuroscience 22: 339–348. 15380833 10.1016/j.ijdevneu.2004.04.002

[6] McMillen BA, Davis BJ, Williams HL, Soderstrom K (2005) Periadolescent nicotine exposure causes heterologous sensitization to cocaine reinforcement. European journal of pharmacology 509: 161–164. 15733551 10.1016/j.ejphar.2005.01.002

[7] Sziraki I, Sershen H, Benuck M, Hashim A, Lajtha A (1999) Differences in Receptor System Participation between Nicotine and Cocaine induced Dopamine Overflow in Nucleus Accumbens. Annals of the New York Academy of Sciences 877: 800–802. 10415708 10.1111/j.1749-6632.1999.tb09326.x

[8] Zernig G, O’Laughlin IA, Fibiger HC (1997) Nicotine and heroin augment cocaine-induced dopamine overflow in nucleus accumbens. European journal of pharmacology 337: 1–10. 9389374 10.1016/s0014-2999(97)01184-9

[9] Carr DB, Sesack SR (2000) Projections from the rat prefrontal cortex to the ventral tegmental area: target specificity in the synaptic associations with mesoaccumbens and mesocortical neurons. The Journal of Neuroscience 20: 3864–3873. 10804226 10.1523/JNEUROSCI.20-10-03864.2000 PMC6772693

[10] Kaddurah-Daouk R, Krishnan KRR (2008) Metabolomics: a global biochemical approach to the study of central nervous system diseases. Neuropsychopharmacology 34: 173–186. 18843269 10.1038/npp.2008.174

[11] Yao JK, Reddy RD (2005) Metabolic investigation in psychiatric disorders. Molecular neurobiology 31: 193–203. 15953821 10.1385/MN:31:1-3:193

[12] Patkar AA, Rozen S, Mannelli P, Matson W, Pae CU, et al (2009) Alterations in tryptophan and purine metabolism in cocaine addiction: a metabolomic study. Psychopharmacology 206: 479–489. 19649617 10.1007/s00213-009-1625-1

[13] Sun L, Li J, Zhou K, Zhang M, Yang J, et al (2013) Metabolomic Analysis Reveals Metabolic Disturbance in the Cortex and Hippocampus of Subchronic MK-801 Treated Rats. PloS one 8: e60598. 23577129 10.1371/journal.pone.0060598 PMC3618452

[14] Duarte I, Ladeirinha A, Lamego I, Gil A, Carvalho L, et al.. (2013) Potential markers of cisplatin treatment response unveiled by NMR metabolomics of human lung cells. Molecular pharmaceutics. 10.1021/mp400335k 24050386

[15] Jung JY, Lee HS, Kang DG, Kim NS, Cha MH, et al (2011) ^1^H-NMR-based metabolomics study of cerebral infarction. Stroke 42: 1282–1288. 21474802 10.1161/STROKEAHA.110.598789

[16] Li Y, Yan G, Zhou J, Bu Q, Deng P, et al (2012) ^1^H Nmr-Based Metabonomics in Brain Nucleus Accumbens and Striatum Following Repeated Cocaine Treatment in Rats. Neuroscience 218: 196–205. 22609933 10.1016/j.neuroscience.2012.05.019

[17] Hu Z, Deng Y, Hu C, Deng P, Bu Q, et al (2012) ^1^H NMR-based metabonomic analysis of brain in rats of morphine dependence and withdrawal intervention. Behavioural Brain Research 231: 11–19. 22391120 10.1016/j.bbr.2012.02.026

[18] Pears MR, Cooper JD, Mitchison HM, Mortishire-Smith RJ, Pearce DA, et al (2005) High resolution 1H NMR-based metabolomics indicates a neurotransmitter cycling deficit in cerebral tissue from a mouse model of Batten disease. Journal of Biological Chemistry 280: 42508–42514. 16239221 10.1074/jbc.M507380200

[19] Salek RM, Colebrooke RE, Macintosh R, Lynch PJ, Sweatman BC, et al (2008) A metabolomic study of brain tissues from aged mice with low expression of the vesicular monoamine transporter 2 (VMAT2) gene. Neurochemical research 33: 292–300. 18041582 10.1007/s11064-007-9542-3

[20] Beckonert O, Keun HC, Ebbels TM, Bundy J, Holmes E, et al (2007) Metabolic profiling, metabolomic and metabonomic procedures for NMR spectroscopy of urine, plasma, serum and tissue extracts. Nature protocols 2: 2692–2703. 18007604 10.1038/nprot.2007.376

[21] Wishart DS, Tzur D, Knox C, Eisner R, Guo AC, et al (2007) HMDB: the human metabolome database. Nucleic acids research 35: D521–D526. 17202168 10.1093/nar/gkl923 PMC1899095

[22] Tian W, Zhao M, Li M, Song T, Zhang M, et al (2012) Reversal of Cocaine-Conditioned Place Preference through Methyl Supplementation in Mice: Altering Global DNA Methylation in the Prefrontal Cortex. PloS one 7: e33435. 22438930 10.1371/journal.pone.0033435 PMC3306398

[23] Itzhak Y, Martin JL, Black MD, Huang PL (1998) The role of neuronal nitric oxide synthase in cocaine-induced conditioned place preference. Neuroreport 9: 2485–2488. 9721919 10.1097/00001756-199808030-00011

[24] Salas R, De Biasi M (2008) Opposing actions of chronic stress and chronic nicotine on striatal function in mice. Neuroscience letters 440: 32–34. 18539390 10.1016/j.neulet.2008.05.038 PMC2536517

[25] Willuhn I, Burgeno LM, Everitt BJ, Phillips PE (2012) Hierarchical recruitment of phasic dopamine signaling in the striatum during the progression of cocaine use. Proceedings of the National Academy of Sciences 109: 20703–20708. 10.1073/pnas.1213460109 PMC3528544 23184975

[26] Bechtholt AJ, Mark GP (2002) Enhancement of cocaine-seeking behavior by repeated nicotine exposure in rats. Psychopharmacology 162: 178–185. 12110995 10.1007/s00213-002-1079-1 PMC2587043

[27] Kelley BM, Middaugh LD (1999) Periadolescent nicotine exposure reduces cocaine reward in adult mice. Journal of addictive diseases 18: 27–39. 10507580 10.1300/J069v18n03_04

[28] Collins SL, Izenwasser S (2004) Chronic nicotine differentially alters cocaine-induced locomotor activity in adolescent vs. adult male and female rats. Neuropharmacology 46: 349–362. 14975690 10.1016/j.neuropharm.2003.09.024

[29] Jutkiewicz EM, Nicolazzo DM, Kim MN, Gnegy ME (2008) Nicotine and amphetamine acutely cross-potentiate their behavioral and neurochemical responses in female Holtzman rats. Psychopharmacology 200: 93–103. 18566803 10.1007/s00213-008-1159-y PMC8009032

[30] Dalley JW, Fryer TD, Brichard L, Robinson ESJ, Theobald DEH, et al (2007) Nucleus accumbens D2/3 receptors predict trait impulsivity and cocaine reinforcement. Science Signalling 315: 1267–1270. 10.1126/science.1137073 PMC1892797 17332411

[31] Maze I, Covington HE III, Dietz DM, LaPlant Q, Renthal W, et al (2010) Essential role of the histone methyltransferase G9a in cocaine-induced plasticity. Science Signalling 327: 213–216. 10.1126/science.1179438 PMC2820240 20056891

[32] Mansvelder HD, McGehee DS (2000) Long-term potentiation of excitatory inputs to brain reward areas by nicotine. Neuron 27: 349–357. 10985354 10.1016/s0896-6273(00)00042-8

[33] Wooltorton JR, Pidoplichko VI, Broide RS, Dani JA (2003) Differential desensitization and distribution of nicotinic acetylcholine receptor subtypes in midbrain dopamine areas. The journal of neuroscience 23: 3176–3185. 12716925 10.1523/JNEUROSCI.23-08-03176.2003 PMC6742341

[34] Pidoplichko VI, Noguchi J, Areola OO, Liang Y, Peterson J, et al (2004) Nicotinic cholinergic synaptic mechanisms in the ventral tegmental area contribute to nicotine addiction. Learning & memory 11: 60–69. 14747518 10.1101/lm.70004 PMC321315

[35] Gass JT, Olive MF (2008) Glutamatergic substrates of drug addiction and alcoholism. Biochemical pharmacology 75: 218–265. 17706608 10.1016/j.bcp.2007.06.039 PMC2239014

[36] Kalivas PW, O’Brien C (2007) Drug addiction as a pathology of staged neuroplasticity. Neuropsychopharmacology 33: 166–180. 17805308 10.1038/sj.npp.1301564

[37] Uys JD, LaLumiere RT (2008) Glutamate: the new frontier in pharmacotherapy for cocaine addiction. CNS & Neurological Disorders-Drug Targets 7: 482–491. 19128205 10.2174/187152708786927868

[38] McFarland K, Lapish CC, Kalivas PW (2003) Prefrontal glutamate release into the core of the nucleus accumbens mediates cocaine-induced reinstatement of drug-seeking behavior. The Journal of Neuroscience 23: 3531–3537. 12716962 10.1523/JNEUROSCI.23-08-03531.2003 PMC6742291

[39] Mao D, Gallagher K, McGehee DS (2011) Nicotine Potentiation of Excitatory Inputs to Ventral Tegmental Area Dopamine Neurons. The Journal of Neuroscience 31: 6710–6720. 21543600 10.1523/JNEUROSCI.5671-10.2011 PMC3118498

[40] Gao M, Jin Y, Yang K, Zhang D, Lukas RJ, et al (2010) Mechanisms involved in systemic nicotine-induced glutamatergic synaptic plasticity on dopamine neurons in the ventral tegmental area. The Journal of Neuroscience 30: 13814–13825. 20943922 10.1523/JNEUROSCI.1943-10.2010 PMC2995497

[41] Rothman RB, Blough BE, Baumann MH (2006) Dual dopamine-5-HT releasers: potential treatment agents for cocaine addiction. Trends in pharmacological sciences 27: 612–618. 17056126 10.1016/j.tips.2006.10.006

[42] Chih CP, Roberts EL (2003) Energy Substrates for Neurons During Neural Activity: A Critical Review of the Astrocyte-Neuron Lactate Shuttle Hypothesis. Journal of Cerebral Blood Flow & Metabolism 23: 1263–1281. 14600433 10.1097/01.WCB.0000081369.51727.6F

[43] Ivanov A, Zilberter Y (2011) Critical State of Energy Metabolism in Brain Slices: The Principal Role of Oxygen Delivery and Energy Substrates in Shaping Neuronal Activity. Frontiers in Neuroenergetics 3. 10.3389/fnene.2011.00009 PMC3247678 22232599

[44] Debora E, Lila O, Allain B, Carlos H, Gabriel S, et al (2011) A palatable hyperlipidic diet causes obesity and affects brain glucose metabolism in rats. Lipids in Health and Disease 16: 18–20. 10.1186/1476-511X-10-168 PMC3198928 21943199

[45] Campfield LA, Smith FJ (1986) Functional coupling between transient declines in blood glucose and feeding behavior: temporal relationships. Brain research bulletin 17: 427–433. 3768746 10.1016/0361-9230(86)90250-9

[46] Ritter RC, Slusser PG, Stone S (1981) Glucoreceptors controlling feeding and blood glucose: location in the hindbrain. Science 213: 451–452. 6264602 10.1126/science.6264602

[47] Canis M, Mack B, Gires O, Maurer MH, Kuschinsky W, et al (2009) Increased densities of monocarboxylate transport protein MCT1 after chronic administration of nicotine in rat brain. Neuroscience research 64: 429–435. 19433117 10.1016/j.neures.2009.04.017

[48] Buccafusco J, Jackson W, Terry A, Marsh K, Decker M, et al (1995) Improvement in performance of a delayed matching-to-sample task by monkeys following ABT-418: a novel cholinergic channel activator for memory enhancement. Psychopharmacology 120: 256–266. 8524972 10.1007/BF02311172

[49] Levin ED, Chen E (2004) Nicotinic involvement in memory function in zebrafish. Neurotoxicology and teratology 26: 731–735. 15451037 10.1016/j.ntt.2004.06.010

[50] Halestrap AP, Price NT (1999) The proton-linked monocarboxylate transporter (MCT) family: structure, function and regulation. Biochemical Journal 343: 281–299. 10510291 PMC1220552

[51] Lan M, McLoughlin G, Griffin J, Tsang T, Huang J, et al (2008) Metabonomic analysis identifies molecular changes associated with the pathophysiology and drug treatment of bipolar disorder. Molecular Psychiatry 14: 269–279. 18256615 10.1038/sj.mp.4002130

[52] Zhang X, Liu H, Wu J, Liu M, Wang Y (2009) Metabonomic alterations in hippocampus, temporal and prefrontal cortex with age in rats. Neurochemistry international 54: 481–487. 19428792 10.1016/j.neuint.2009.02.004

[53] Pellerin L (2003) Lactate as a pivotal element in neuron–glia metabolic cooperation. Neurochemistry international 43: 331–338. 12742077 10.1016/s0197-0186(03)00020-2

[54] Khan AR, Rana P, Devi MM, Chaturvedi S, Javed S, et al (2010) Nuclear magnetic resonance spectroscopy-based metabonomic investigation of biochemical effects in serum of γ-irradiated mice. International journal of radiation biology 87: 91–97. 21087167 10.3109/09553002.2010.518211

[55] Macallan DC (1999) Malnutrition in tuberculosis. Diagnostic microbiology and infectious disease 34: 153–157. 10354866 10.1016/s0732-8893(99)00007-3

[56] Macallan D, McNurlan M, Kurpad A, De Souza G, Shetty P, et al (1998) Whole body protein metabolism in human pulmonary tuberculosis and undernutrition: evidence for anabolic block in tuberculosis. Clinical science 94: 321–331. 9616267 10.1042/cs0940321

[57] Levin L, Gevers W, Jardine L, De Guel F, Duncan E (1983) Serum amino acids in weight-losing patients with cancer and tuberculosis. European Journal of Cancer and Clinical Oncology 19: 711–715. 6683643 10.1016/0277-5379(83)90002-0

[58] Barnwell S, Sastry B (1984) Inhibition of the uptake of amino acids in human placental villus by nicotine, cocaine and morphine. Springer US: 101–120.

[59] Sreekumar A, Poisson LM, Rajendiran TM, Khan AP, Cao Q, et al (2009) Metabolomic profiles delineate potential role for sarcosine in prostate cancer progression. Nature 457: 910–914. 19212411 10.1038/nature07762 PMC2724746

